# Clinical factors associated with growth and neoantigen reactivity of tumor infiltrating lymphocytes from metastatic epithelial cancers

**DOI:** 10.1007/s00262-025-04091-3

**Published:** 2025-06-19

**Authors:** Alexandra M. Gustafson, Aaron J. Dinerman, Kyle J. Hitscherich, Maria R. Parkhurst, Hyunmi Halas, Donald E. White, Chuong D. Hoang, Jonathan M. Hernandez, Mei Li M. Kwong, Nicholas D. Klemen, Steven A. Rosenberg, Stephanie L. Goff, James C. Yang

**Affiliations:** 1 Surgery Branch, National Cancer Institute, National Institutes of Health, Bethesda, MD USA; 2 Surgical Oncology Program, National Cancer Institute, National Institutes of Health, Bethesda, MD USA; 3 Thoracic Surgery Branch, National Cancer Institute, National Institutes of Health, Bethesda, MD USA

**Keywords:** Tumor infiltrating lymphocytes, Metastatic solid tumors, Adoptive cell transfer

## Abstract

**Background:**

Adoptive cell therapy with tumor infiltrating lymphocytes is FDA approved for metastatic melanoma. TIL from patients with melanoma and factors relating to growth and reactivity have been studied; however, this has not been explored in patients with epithelial cancers.

**Patients and methods:**

Metastatic epithelial tumors resected for TIL growth from 2014 to 2023 were analyzed. Two hundred and ninety-one operations were performed to collect TIL for potential treatment. Of these, 263 harvests were each processed for up to 24 individual fragment cultures and screened for neoantigen recognition of the expressed products of cancer mutations. Patient and tumor characteristics were collected. Endpoints were growth (defined as more than half of all fragment cultures expanded for viable cryopreservation) and patient-specific neoantigen reactivity (release of interferon-γ in cocultures measured by ELISpot and 4-1BB upregulation on flow cytometry).

**Results:**

TIL fragments reached adequate growth for screening by 21 days. Metastatic resections from lung were more likely to grow TIL than all other resection sites combined (95%, *p* = 0.0011), while hepatic resections were less likely to grow (69%, *p* < 0.0001). One hundred and thirty-five patients (51%) had highly reactive TIL, 68 (26%) had weakly reactive TIL, and 60 (23%) had TIL with no neoantigen reactivity. Patients with prior exposure to immune checkpoint blockade therapy were less likely to have highly reactive TIL (*p* = 0.0325). Metastatic resection site impacted TIL reactivity against neoantigens, with those harvested from the lung more likely to show any reactivity (83%, *p* = 0.0180), as well as high reactivity (59%, *p* = 0.0066).

**Conclusions:**

Prior immune checkpoint therapies reduced the likelihood of having highly reactive TIL. Neoantigen reactivity was more common in TIL from thoracic resections versus other sites. Conversely, hepatic lesions yielded TIL less likely to grow and with less reactivity. These results contribute to improved strategies for sequencing TIL with other therapies and planning TIL harvests for patients with epithelial cancers.

**Supplementary Information:**

The online version contains supplementary material available at 10.1007/s00262-025-04091-3.

## Background

In 2024, it is estimated that over 2 million patients will be diagnosed with cancer, with lung, breast, prostate, pancreas and colorectal cancer accounting for over half of all cancer-related deaths [1]. While surgery, chemotherapy, and radiation remain the mainstays of treatment, immunotherapy has found a role in the treatment of certain types of cancers [2–5].

Adoptive cell transfer (ACT) with tumor infiltrating lymphocytes (TIL) is able to mediate durable tumor regression in patients with metastatic melanoma and has recently obtained FDA approval for use in advanced melanoma [6]. While less frequently seen than in melanoma, TIL do have the ability to cause tumor shrinkage in common epithelial cancers [7–9] (Supplemental Fig. 1). Past analysis of tumors resected for the purpose of TIL generation in patients with metastatic melanoma revealed factors that correlate with successful growth and reactivity of TIL [10]. Not only do melanoma tumors harbor a higher number of mutations, but systemic therapy for melanoma is fundamentally different than that of epithelial cancers, making it difficult to extrapolate which factors truly affect TIL in this population. For example, it is not uncommon for patients with metastatic breast cancer to receive up to 10 lines of therapy [11]. Whether multiple prior lines of cytotoxic therapy affect TIL generation and reactivity, and to what degree, is unknown. The aim of this study is to evaluate what impact prior therapies and tumor features have on the ability to grow reactive TIL in patients with metastatic epithelial cancers.

## Methods

Patients with metastatic epithelial cancer who had surgical resections at our institution between August 2014 and September 2023 for the purpose of TIL generation were reviewed. All patients were included as part of NCI protocol NCT00068003. Patient demographic information was collected from medical records, with a focus on past systemic therapy for the treatment of cancer. Variables recorded include radiation (bone vs non-bone), type of chemotherapy, duration of cumulative chemotherapy, and receipt of immune checkpoint blockade (IBD; i.e., PD-1, PD-L1, and CTLA-4 inhibitors). Using operative notes and pathology reports, site of tumor resection was obtained, as well as total number of tumors in the specimen and maximum diameter of largest lesion. Tumor resections which were solely for research purposes and those performed after prior adoptive cell transfer were excluded.

**Fig. 1:**
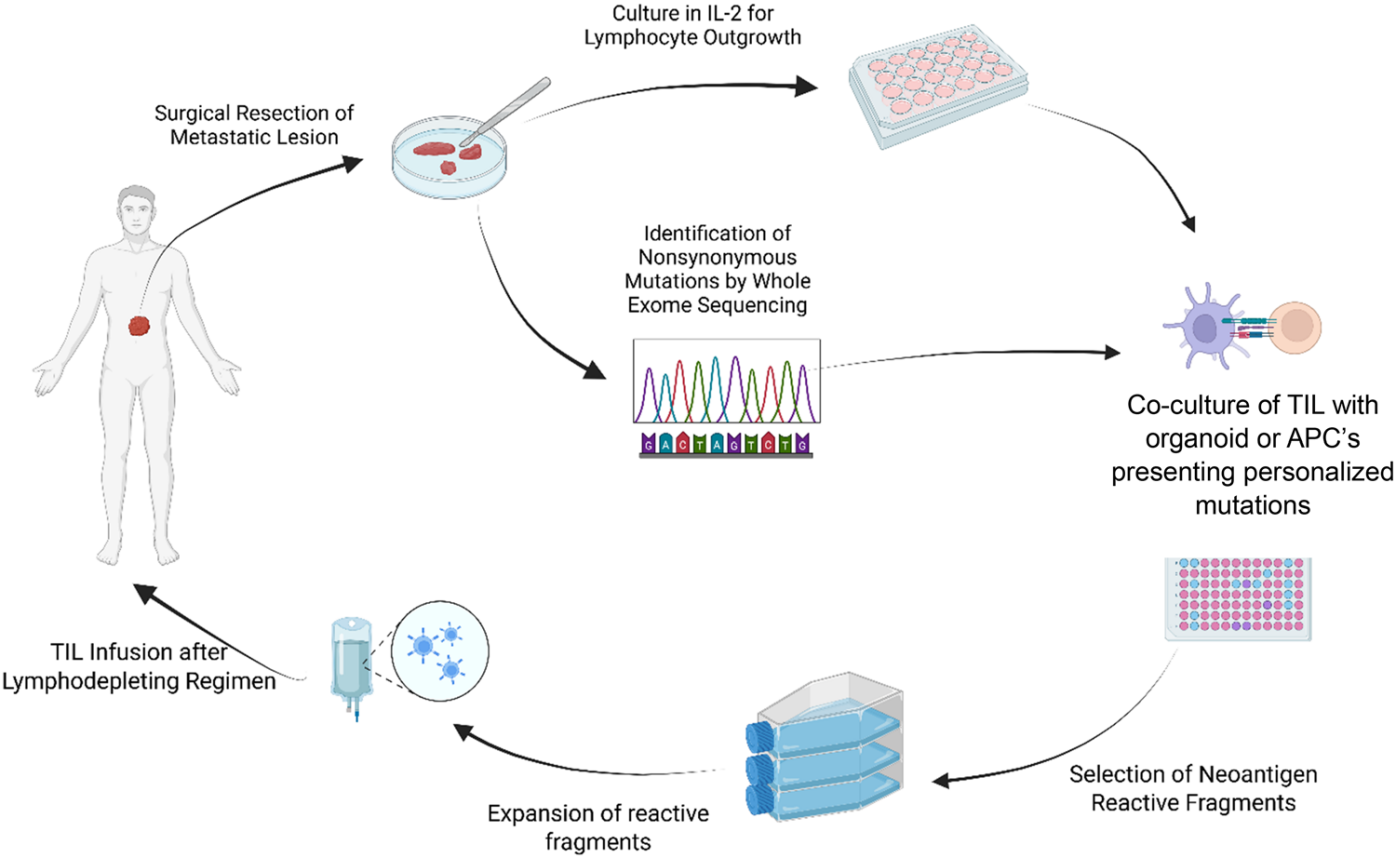
Schema of TIL growth and neoantigen screening in metastatic epithelial cancers Created in BioRender. Dinerman, A. (2025) https://BioRender.com/jwlc2su

TIL from tumor specimens were grown as previously described (Fig. 1) [12]. Briefly, tumors are divided into up to 24 fragments and plated individually in a 24 well plate with T cell medium containing high-dose interleukin-2 to allow for outgrowth of lymphocytes. TIL growth was defined as at least half of all starting fragments reaching four confluent wells in a 24 well plate. Four wells represent an adequate amount of cells to screen for neoantigen reactivity and then expand for clinical administration if reactive TIL are identified. All TIL fragment cultures showing sufficient growth are maintained separately and screened against the expressed products of individual cancer mutations as previously described [13]. In brief, all nonsynonymous point mutations identified by whole exome sequencing are encoded by 25 codon minigenes which include the normal genetic flanking sequences around the mutation. The frame-shifted regions created by insertions and deletions are encoded in their entirety, and 10–15 of these minigenes are concatenated into tandem minigenes (TMG), which are then transfected as RNA into autologous dendritic cells to create an antigen presenting cell displaying the patient’s tumor-specific mutations. In addition, mutations are also expressed as synthetic 25-mer peptides (with the variant amino acid in the middle) which are then pulsed in pools of 10–20 peptides onto autologous dendritic cells which serve as an alternate method for displaying tumor mutations on antigen presenting cells. Both of these tumor cell surrogate targets are cocultured with the patient’s TIL to detect neoantigen reactivity.

**Fig. 2:**
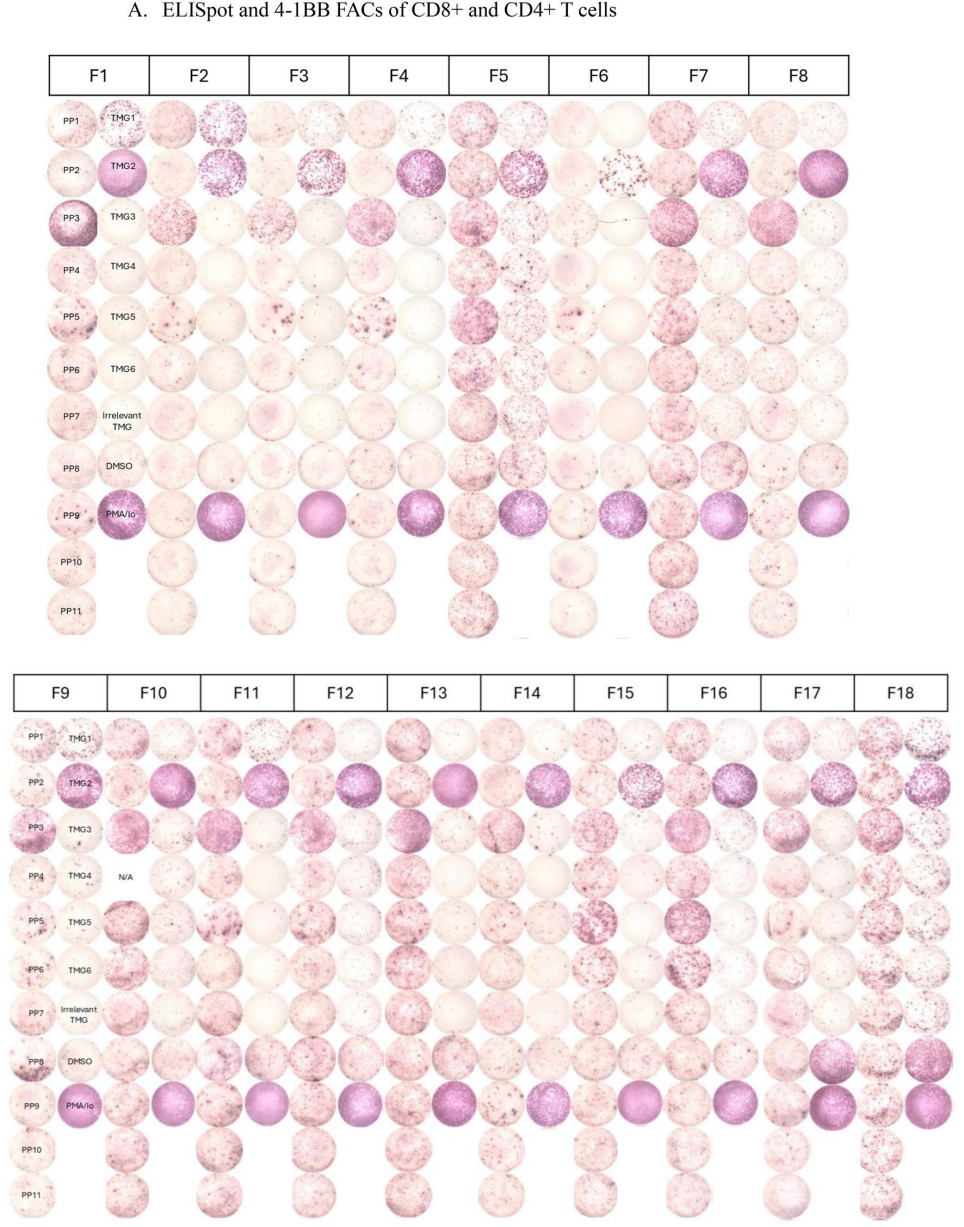

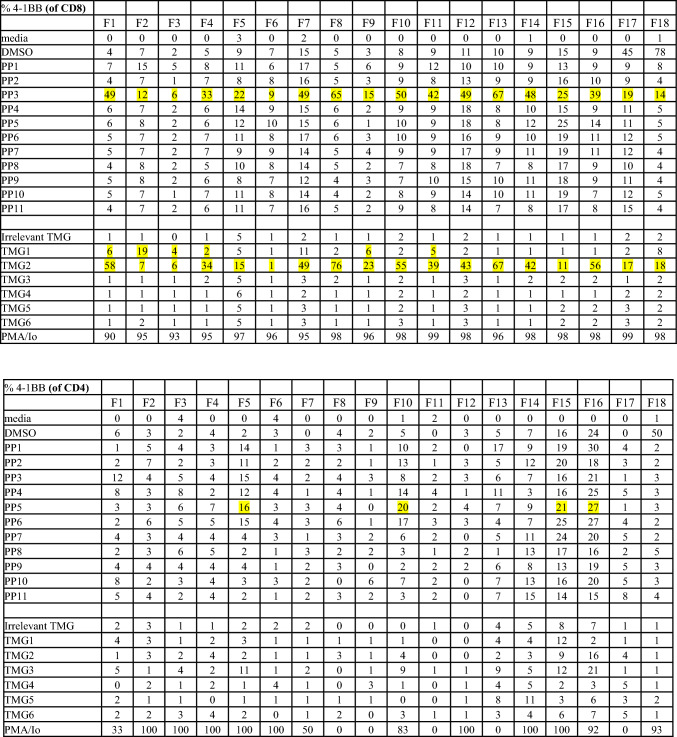

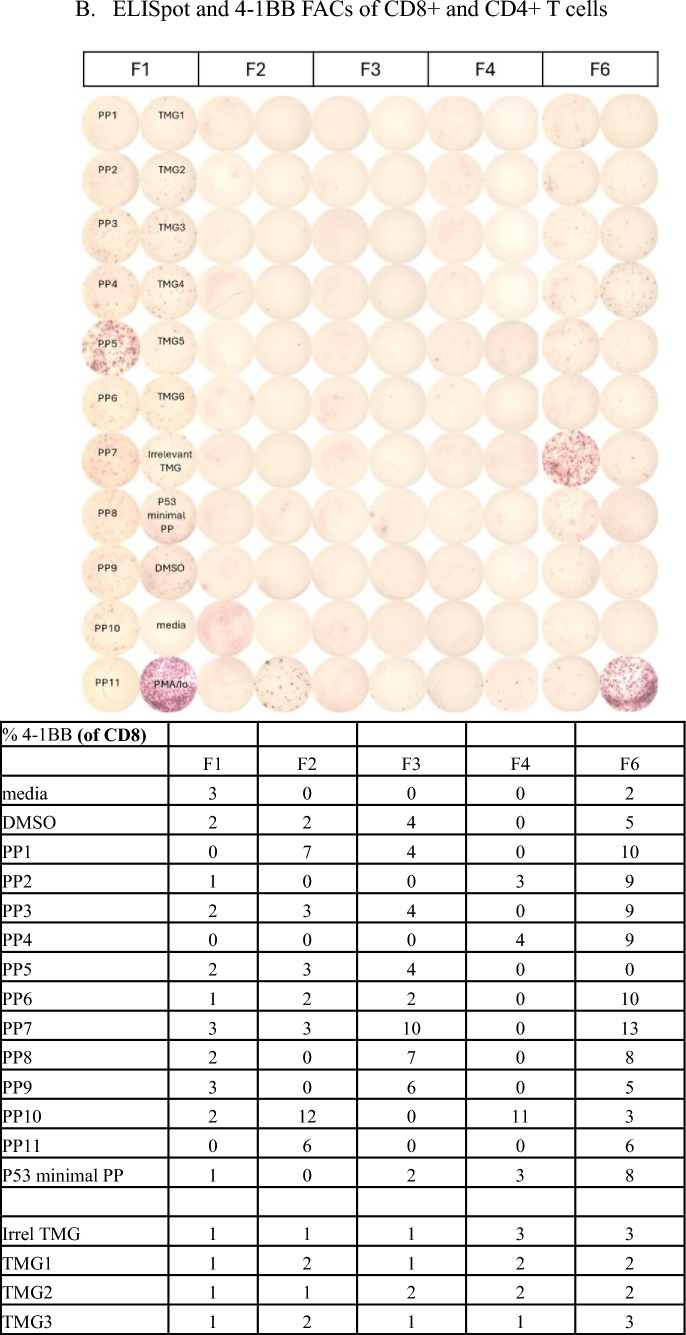

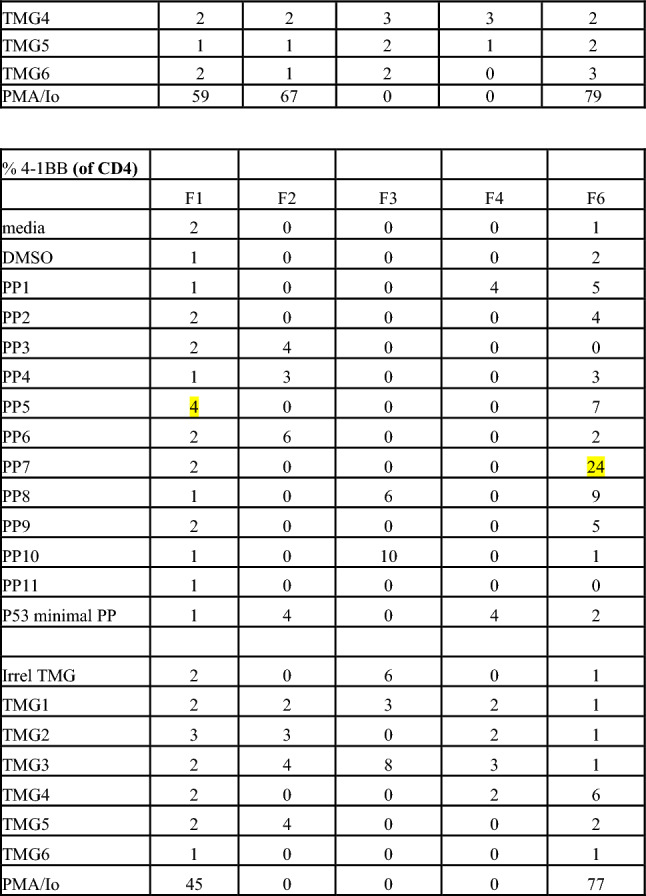

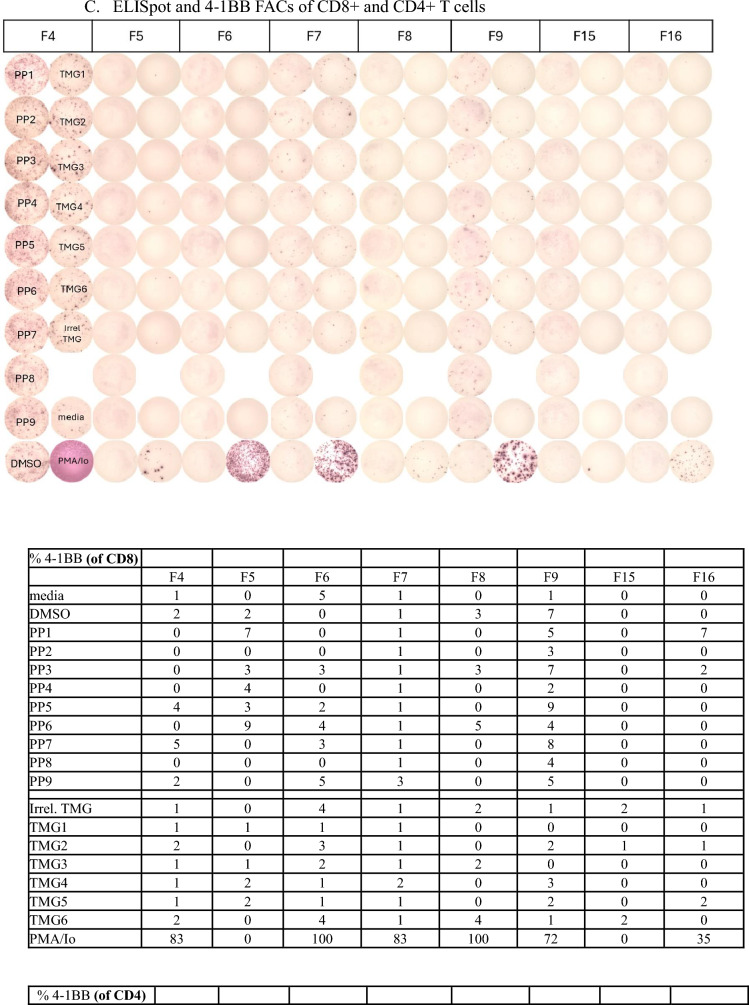

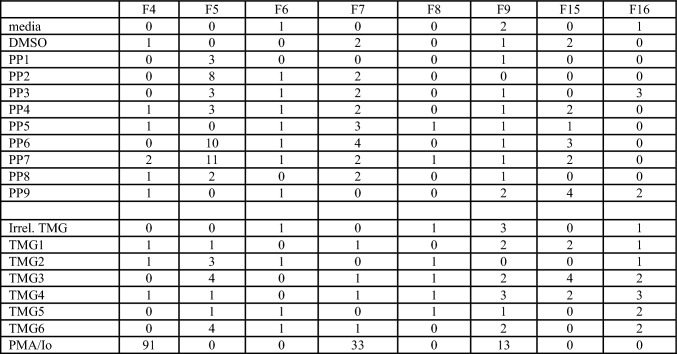
Examples of highly reactive, weakly reactive, and negative TIL screens. **A** Images of IFN gamma ELISpot plate and percentage 4-1BB expression by FACS. 18 TIL fragment cultures (columns F1 through F18) from a patient with colorectal cancer with highly reactive TIL harvested from a lung lesion were cocultured with their dendritic cells, either electroporated with RNA from their tandem minigenes (TMG) encoding all tumor mutations or pre-incubated with pools of synthetic peptides (PP) containing all tumor mutations. Multiple fragment cultures react with mutant TMG and PP, notably TMG1, TMG2, and PP3. **B** Five TIL fragment cultures from a patient with colorectal cancer with weakly reactive TIL harvested from in intra-abdominal lesion (F1 is reactive with PP5 and F6 is reactive with PP7). Fourteen of 24 plated fragments grew to sufficient number for testing (not shown, additional negative fragments). **C** Eight TIL fragment cultures from a hepatic lesion from a patient with colorectal cancer. Only eight of 24 plated fragments grew to sufficient number for testing. No recognition of TMG’s or PP’s seen. TMG: Tandem Mini Gene, PP: Peptide Pool, Irrel TMG: Irrelevant TMG as negative control, DMSO: Peptide free solvent as negative peptide control, PMA/Io: PMA + Ionomycin as a maximum positive control, N/A: not available. Highlighted 4-1BB values correspond with qualitatively positive ELISpot wells

Reactivity of TIL is determined by specific interferon-γ release in cocultures measured by ELISpot and 4-1BB expression measured via flow cytometry. Patients with 2 or more TIL fragments reactive against neoantigens are considered to have reactive TIL and are eligible for ACT treatment. Cultures with qualitatively positive ELISpots and > 20% 4-1BB upregulation as compared to negative controls were deemed reactive. Patients with low levels of interferon-γ release or low frequency of 4-1BB expression in less than 2 fragment cultures are considered to have weakly reactive TIL (insufficient for clinical administration). These patient’s TIL frequently undergo further analysis to identify individual T cell receptors recognizing defined neoantigens from these reactive fragments. Finally, patients with no specific increase in interferon-γ or 4-1BB upregulation are considered to have nonreactive TIL. Examples of highly reactive, weakly reactive, and nonreactive TIL screens are shown in Fig. 2.

We sought to analyze the data for highly reactive TIL—defined as those patients with sufficient reactivity to treat with TIL, as well as any reactive TIL—combining patients with robust TIL and those with weakly reactive TIL. Analysis of TIL reactivity is reported in terms of clinical significance (comparing highly reactive TIL to weakly and nonreactive TIL) and scientific significance (comparing highly reactive and weakly reactive TIL to nonreactive TIL).

Statistical analysis was performed with GraphPad Prism version 10.1.1 (GraphPad Software, San Diego, CA). Fisher’s exact test was used to assess association between dichotomous variables, chi-square test for trend was used for cumulative chemotherapy exposure, and Mann–Whitney test was used for TIL size and number of tumors.

## Results

A total of 291 operations were performed for the purpose of TIL generation over a nine-year period. The most common primary site of cancer in all patients was colorectal (47%, Table 1), followed by breast (22%) and pancreatic (11%). The most common site of tumor procurement was lung (53%, Table 1), followed by liver (19%) and lymph node (13%). The median length of hospital stay for TIL harvest surgeries was 2 days (Supplemental Table 1). Four patients (1%) had complications from surgery requiring additional intervention (Supplemental Table 1). These patients required additional drainage tubes for bile leak after hepatic resection or pneumothorax after thoracic resection.

**Table 1:** Patient demographics and tumor characteristics

		Percentage of total (%)
Total	291	
*Patient demographics*		
Male	112	39
Age median (IQR)	51 (45–59)	
**ECOG**		
0	238	82
1	53	18
**Primary cancer diagnosis**		
Colorectal	137	47
Breast	65	22
Pancreatic	32	11
Cholangiocarcinoma	18	6
Gastroesophageal	12	4
Non-small cell lung carcinoma	9	3
Ovarian	7	3
Other	11	4
*Tumor characteristics*		
**Metastasectomy site**		
Thoracic	155	53
Hepatic	54	19
Lymph node	37	13
Intraperitoneal	24	8
Soft tissue	21	7
Median number of tumors resected (IQR)	2 (1–3)	
Median diameter of largest tumor resected in cm (IQR)	2.5 (1.9–3.2)	

Adequate TIL growth was achieved in 89% of all resections. Patients with a diagnosis of pancreatic cancer were less likely to grow viable TIL compared to all other types of cancer (71% vs 91%, *p* = 0.003, Table 2). The number of tumors resected per patient ranged from 1 to 10, with a median of 2. Maximum diameter of the largest tumor resected ranged from 0.5 to 11 cm, with a median diameter of 2 cm. Neither number of tumors resected nor tumor size affected TIL growth. Over half of all resections were of pulmonary metastases (Table 1), followed by hepatic resections and lymph node harvests. Metastasectomies of hepatic tumors were less likely to generate sufficient TIL compared to all other sites (69% vs. 93%, *p* < 0.0001), whereas resections of pulmonary tumors were more likely to generate TIL (95% vs 82%, *p* = 0.001).

**Table 2:** TIL growth data

	Growth Yes 259 (89%)	Growth No 32 (11%)	Significance *p* value
*Patient characteristics*			
**Sex**			
Male (112–38%)	97 (87%)	15 (13%)	0.3378
Female (179–62%)	162 (91%)	17 (9%)	
Primary cancer diagnosis			
Colorectal (138–47%)	127 (92%)	11 (8%)	0.1353
Breast (65–22%)	59 (91%)	6 (9%)	0.8220
Pancreas (31–11%)	22 (71%)	9 (29%)	**0.0028**
Cholangiocarcinoma (18–6%)	17 (94%)	1 (6%)	0.7038
Other (39–13%)	34 (87%)	5 (13%)	
**5FU exposure**			
Yes (223–77%)	200 (90%)	23 (10%)	0.5096
No (68–23%)	59 (87%)	9 (13%)	
**Platinum-based chemoexposure**			
Yes (235–81%)	211 (90%)	24 (10%)	0.3253
No (56–19%)	48 (86%)	8 (14%)	
**Chemotherapy within 6 weeks of TIL harvest**			
Yes (59–20%)	52 (88%)	7 (12%)	0.8168
No (232–80%)	207 (89%)	25 (11%)	
**Cumulative chemoexposure**			
None (11–4%)	10 (91%)	1 (9%)	0.5069
0–3 months (8–3%)	6 (75%)	2 (2%)	
3.1–6 months (42–14%)	37 (88%)	5 (12%)	
6.1–12 months (69–24%)	61(88%)	8 (12%)	
> 12 months (161–55%)	145 (90%)	16 (10%)	
**Bone radiation exposure**			
Yes (24–8%)	22 (92%)	2 (8%)	> 0.9999
No (267–92%)	237 (89%)	30 (11%)	
**Immune checkpoint blockade exposure**			
Yes (50–17%)	42 (84%)	8 (16%)	0.2181
No (241–83%)	217 (90%)	24 (10%)	
*Tumor characteristics*			
**Harvest site**			
Liver (54–19%)	37 (69%)	17 (31%)	**< 0.0001**
Lymph Node (37–13%)	35 (95%)	2 (5%)	0.3965
Intraperitoneal (24–8%)	20 (83%)	4 (17%)	0.3151
Thoracic (155–53%)	147 (95%)	8 (5%)	**0.0011**
Soft Tissue (21–7%)	20 (95%)	1 (5%)	0.4882
Median tumor size in cm (IQR)	2 (1–3)	2 (1.25–3)	0.8983
Median number of tumors (IQR)	2 (1–3)	2 (1.25–3)	0.5219
Median growth time (days)	20 (17–25)	Not Reached	

*IQR* interquartile range

The majority of patients had received a Fluorouracil (5FU) or platinum-based chemotherapy regimen; this had no impact on ability to generate viable TIL (*p* = 0.51 and 0.33, respectively). While over half of all patients had received greater than 12 months of chemotherapy, cumulative duration of chemotherapy exposure did not affect TIL growth (*p* = 0.51). Patients who received chemotherapy within 6 weeks of TIL harvest were just as likely as those with more remote chemotherapy exposure to produce viable TIL (p = 0.82). Additionally, prior radiation and receipt of ICB had no impact on TIL growth (*p* > 0.99 and *P* = 0.22, respectively).

Of the 291 resections, 263 TIL samples were screened for neoantigen recognition. The remaining TIL were not tested due to either poor growth, or patient-specific factors such as treatment on a different protocol or progressive disease making them ineligible for treatment. Some TIL cultures did not fulfill our minimal growth criteria but still had sufficient T cells to perform screening, and thus are included in reactivity analysis. One hundred and thirty-five (51%) patients had highly reactive TIL, 68 (23%) had weakly reactive TIL, and 60 (23%) had no reactivity. Patients with a primary diagnosis of colorectal cancer were more likely to have neoantigen reactive TIL versus all others, when considering high reactivity or any reactivity (60% vs 44%, *p* = 0.01 and 84% vs 71%, *p* = 0.02; Table 3). Similar to TIL growth, receipt of prior 5FU and platinum-based chemotherapy regimens did not affect TIL reactivity. Additionally, cumulative chemotherapy exposure had no impact on TIL reactivity. Proximity to most recent chemotherapy exposure did not affect TIL growth or reactivity. Patients with chemotherapy within 6 weeks of harvest were just as likely as patients with more remote chemotherapy exposure to generate highly reactive TIL (47% vs 52%, *p* = 0.53; Table 3). Patients with prior bone irradiation had a higher frequency of TIL with undetectable reactivity compared to the group without prior radiation (39% vs 21%, *p* = 0.07), but this analysis is limited by small sample size. Patients who received prior treatment with ICB were less likely to generate highly reactive TIL compared to those who were ICB naive (36% vs 54%, *p* = 0.03). The distribution of patients who received ICB was largely representative of the entire group’s histology without significant differences, although patients with NSCLC and gastroesophageal cancer received ICB slightly more frequently.

**Table 3:** TIL reactivity data

	Highly reactive 135 (51%)	Weakly reactive 68 (26%)	Nonreactive 60 (23%)	Clinical significance (highly reactive vs weakly reactive plus nonreactive) *p* value	Scientific significance (highly reactive plus weakly reactive vs. nonreactive) *p* value
*Patient characteristics*					
**Sex**					
Male (98–37%)	55 (56%)	22 (22%)	21 (22%)	0.2523	0.7618
Female (165–63%)	80 (48%)	46 (28%)	39 (24%)		
**Primary cancer diagnosis**					
Colorectal (124–47%)	74 (60%)	30 (24%)	20 (16%)	**0.0134**	**0.0183**
Breast (50–29%)	22 (44%)	14 (28%)	14 (28%)	0.2736	0.3513
Pancreatic (26–10%)	10 (38%)	7 (27%)	9 (35%)	0.2153	0.1426
Cholangiocarcinoma (15–6%)	9 (60%)	2 (13%)	4 (27%)	0.5985	0.7524
**5FU exposure**					
Yes (199–76%)	102 (51%)	56 (28%)	41 (21%)	> 0.9999	0.1699
No (64–24%)	33 (51%)	12 (19%)	19 (30%)		
**Platinum-based chemoexposure**					
Yes (211–80%)	113 (53%)	54 (26%)	44 (21%)	0.1648	0.1413
No (52–20%)	22 (42%)	14 (27%)	16 (31%)		
**Chemotherapy within 6 weeks of TIL harvest**					
Yes (51–19%)	24 (47%)	14 (27%)	13 (26%)	0.5348	0.5832
No (212–81%)	111 (52%)	54 (26%)	47 (22%)		
**Cumulative chemoexposure**					
None (11–4%)	6 (55%)	3 (27%)	2 (18%)	0.7918	0.6119
0–3 months (7–3%)	4 (57%)	2 (29%)	1 (14%)		
3.1–6 months (41–16%)	20 (49%)	11 (27%)	10 (24%)		
6.1–12 months (62–24%)	29 (47%)	20 (32%)	13 (21%)		
> 12 months (142–54%)	76 (53.5%)	32 (22.5%)	34 (24%)		
**Bone radiation exposure**					
Yes (23–9%)	9 (39%)	5 (22%)	9 (39%)	0.2763	0.0670
No (240–91%)	126 (53%)	63 (26%)	51 (21%)		
**immune checkpoint blockade exposure**					
Yes (44–17%)	16 (36%)	15 (34%)	13 (30%)	**0.0325**	0.2433
No (219–83%)	119 (54%)	53 (24%)	47 (22%)		
*Tumor characteristics*					
**Harvest site**					
Liver (48–18%)	18 (37.5%)	12 (25%)	18 (37.5%)	**0.0385**	**0.0124**
Lymph node (32–12%)	14 (44%)	10 (31%)	8 (25%)	0.4510	0.8223
Intraperitoneal (20–8%)	7 (35%)	7 (35%)	6 (30%)	0.1637	0.4137
Thoracic (142–87%)	84 (59%)	34 (24%)	24 (17%)	**0.0066**	**0.0180**
Soft tissue (21–8%)	12 (57%)	5 (24%)	4 (19%)	0.6527	0.7918
Median tumor size (IQR)	2.4 (2–3)	2.5 (1.8–3.8)	2.5 (2–3.5)	0.2826	0.4382
Median number of tumors (IQR)	2 (1–3)	2 (1–3)	2 (1–3)	0.2022	0.2282
Median growth time (days)	19 (16–23)	21 (19–26)	20 (16–26)	**0.0086**	0.3862

*IQR* interquartile range

Metastasectomy site was associated with TIL reactivity. Hepatic harvests were less likely to generate highly reactive TIL and any reactivity (38% vs 54%, *p* = 0.04; 63% vs 80%, *p* = 0.01), while the reverse was seen with thoracic harvests versus all other sites (59% vs 42% highly reactive, *p* = 0.01; 83% vs 70% any reactivity, *p* = 0.02). Metastasectomies of soft tissue lesions yielded highly reactive TIL at a frequency similar to thoracic TIL harvests (57%) but this was not significantly higher compared to all other sites (*p* = 0.65). Again, this finding is limited by the small number of patients undergoing soft tissue resections for TIL and the high frequency of thoracic resections. The size and number of tumors resected did not impact TIL reactivity. Finally, those TIL which were highly reactive grew more rapidly and could be screened earlier compared to weakly reactive and nonreactive TIL (median 21 days vs 19 days, *p* = 0.01).

## Discussion

TIL therapy has the potential to provide long-term benefit in select patients with metastatic epithelial cancers; the first step in this treatment is to procure viable and reactive T cells from patients. Our institution has extensive experience in harvesting metastatic lesions from epithelial cancers to isolate TIL. This review of a large, heterogeneous group of patients is an attempt to identify which patient and tumor characteristics increase the likelihood of generating reactive TIL.

While patients with microsatellite-instability-high or mismatch-repair-deficient tumors show improved survival with immune checkpoint blockade [14], all patients in our cohort have microsatellite stable/mismatch-repair proficient cancers. Patients with pancreatic adenocarcinoma were less likely to produce viable TIL compared to other histologies. This is consistent with the heavily desmoplastic nature of pancreatic cancer and the associated immunosuppressive tumor microenvironment [15]. The fibrotic stroma poses a challenge for the infiltration of cytotoxic T cells and thus impacts the number of viable and reactive cells aggregated in the tumor environment. Conversely, patients with colorectal cancer were more likely to have neoantigen reactive TIL.

The choice of metastasectomy site relies primarily on which surgical approach is the least morbid while obtaining sufficient tumor to grow lymphocytes. Beyond this, no clear guidelines exist in deciding which metastatic lesions to harvest for TIL. We found that hepatic lesions were associated with less likelihood of growing viable and reactive TIL. Whether this is a direct cause of the immunosuppressive liver tumor microenvironment, or liver metastatic spread is a negative prognostic marker that indicates aggressive tumor biology with worse TIL growth, is unknown. Review of this cohort of patients revealed that pulmonary metastatic lesions were most likely to harbor viable and reactive TIL, with almost 60% of all patients who underwent pulmonary resections generating reactive TIL sufficient for ACT. One possible explanation for this is the different immune microenvironment inherent to organs of various metastatic sites. Using transcriptomic data to categorize metastatic lesions according to immunogenicity, Garcia-Mulero et al. found that lung metastases exhibited a distinct inflammatory phenotype with increased immune infiltrate, regardless of site of primary tumor [16]. Furthermore, murine studies of pulmonary and hepatic metastases in pancreatic cancer confirmed upregulated immune signaling pathways in the lung and immune-suppressive pathways in the liver [17, 18]. Our findings of increased TIL generation from pulmonary metastases clinically correlate with evidence of differing immune environments in various organs.

Prior chemotherapy and radiation exposure did not affect the ability to grow or generate reactive TIL. Although we did not define the shortest permissible interval from chemotherapy to TIL procurement, it appears that six weeks is not detrimental for regimens commonly used for gastrointestinal cancers. This finding is encouraging since patients with advanced epithelial cancers frequently undergo multiple lines of therapy and present for TIL treatment heavily pre-treated. However, patients who had previously been treated with an ICB were less likely to harbor robustly reactive TIL. This may simply represent a selection bias, in that patients who have progressed through a prior immunotherapy have demonstrated a weak de-novo immune recognition of their tumor. While only 17% of all patients had received a checkpoint inhibitor, new immunotherapies are approved every year for epithelial cancers, and it is expected that more patients will present with prior checkpoint inhibitor exposure. Whether checkpoint inhibitor therapy truly has an effect on TIL repertoire or T cell exhaustion would require a longitudinal cohort study for individuals undergoing checkpoint inhibitor therapy and could serve as a future question of interest.

There are some limitations of this study to take into consideration. It is a retrospective analysis of our experience at a single institution, which may limit its generalizability. Additionally, poor TIL growth does not always portend poor clinical outcomes. There are patients who had slow growth of their TIL but were able to be treated despite only a handful of fragments growing TIL. Additionally, although hepatic lesions demonstrated less likelihood of generating viable and reactive TIL, some patients received reactive TIL from a liver metastasis and experienced tumor shrinkage after receiving these TIL. While this is not frequently the case, it is still possible, and thus patients with exclusively hepatic metastases should not be excluded from TIL treatment. The number of treated and responding patients on the current trial is not sufficient for a robust analysis of correlations with objective responses. Therefore, this study is confined to analysis of the first component of tumor rejection by TIL, which is specific immunological recognition of tumor neoantigens.

This analysis is intended for use in planning resections and TIL therapy in patients with metastatic epithelial cancers. In the event of multiple sites of metastases, we have shown that resection of pulmonary tumors produces superior TIL from both a growth and reactivity standpoint. While there was a difference between resection sites, reactive TIL was able to be generated from every metastatic site. The field of immunotherapy continues to grow, and although treatment with TIL is extremely personalized and is not always an option for patients, these data may be useful in determining when and from where TIL should be harvested for those patients in which TIL treatment is available.

## Supplementary Information

Below is the link to the electronic supplementary material.

Supplementary file1 (DOCX 480 kb)

## Data Availability

No datasets were generated or analyzed during the current study.
