# Supplementary material for: Clinical factors associated with growth and neoantigen reactivity of tumor infiltrating lymphocytes from metastatic epithelial cancers
Source: Cancer Immunol Immunother. 2025 Jun 19;74(8):244. doi: 10.1007/s00262-025-04091-3 (PMC12179044; doi:10.1007/s00262-025-04091-3)
Supplement: Supplementary file 1 — Supplementary file1 (DOCX 480 kb) [file 262_2025_4091_MOESM1_ESM.docx]

| Pre-Treatment | 11 months Post-Treatment |
| --- | --- |


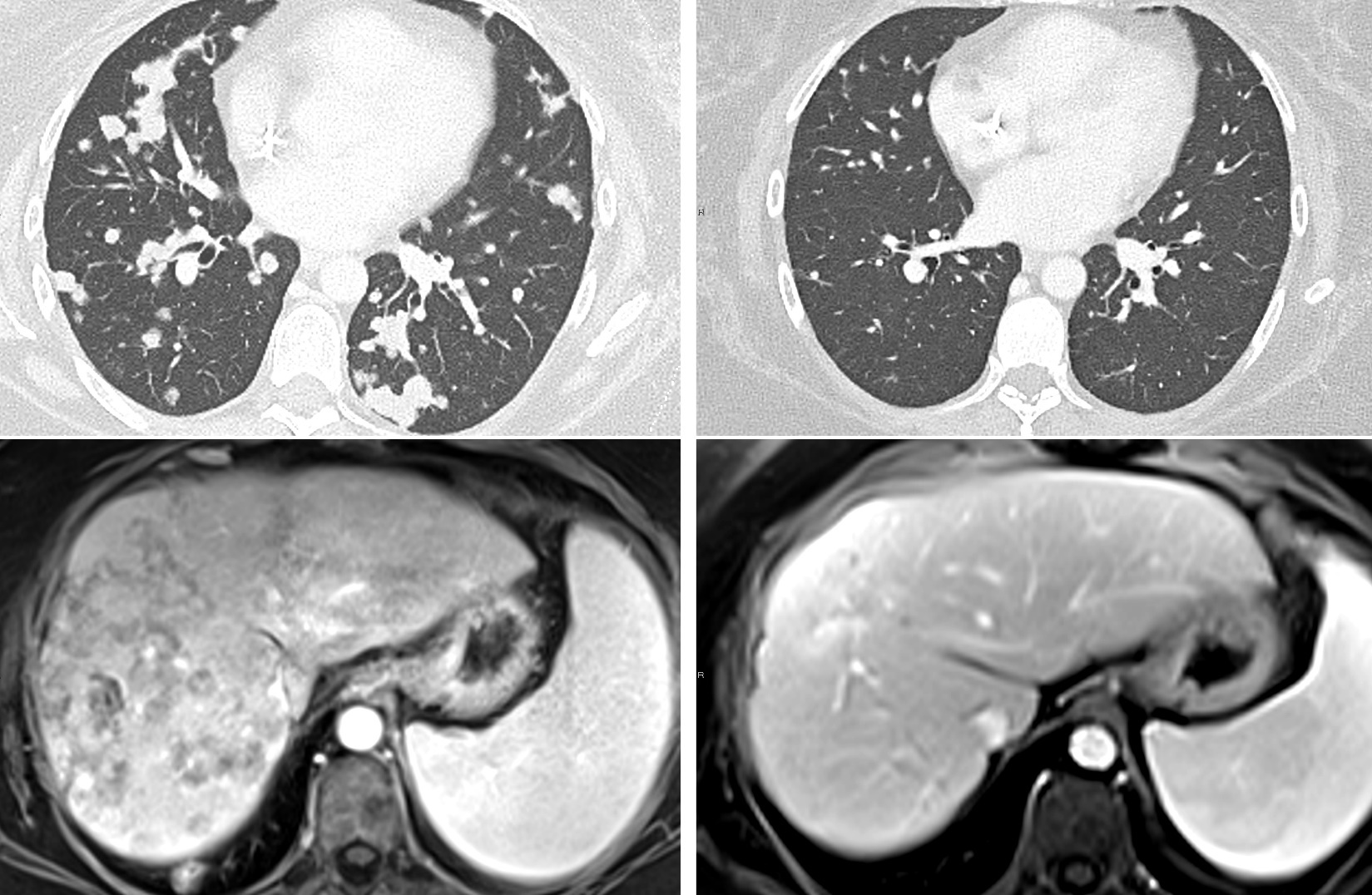
**Supplemental Figure 1:** Chest computed topography and liver magnetic resonance imaging of a patient with colorectal cancer and TIL harvest from a thoracic lesion which had high neoantigen reactivity. Patient with ongoing partial response of 11+ months.

**Supplemental Table 1:** Hospital Length of Stay of Patients during Metastasectomy

*Interventions required were insertion of IR drain for bile leak or pneumothorax

| **Metastectomy Site** | Median Hospital Length of Stay in Days (Range) | Number of Complications requiring Intervention* (%) |
| --- | --- | --- |
| **All Patients** | 2 (0-10) | 4 (1%) |
| Thoracic (n=155) | 2 (0-10) | 1 (1%) |
| Hepatic (n=54) | 3 (1-8) | 3 (6%) |
| Lymph Node (n=37) | 1 (0-6) | 0 (0%) |
| Intraperitoneal (n=24) | 2 (1-8) | 0 (0%) |
| Soft Tissue (n=21) | 1 (0 – 3) | 0 (0%) |
